# Nasal carriage of common bacterial pathogens among healthy kindergarten children in Chaoshan region, southern China: a cross-sectional study

**DOI:** 10.1186/s12887-016-0703-x

**Published:** 2016-09-30

**Authors:** Hui Pan, Binglin Cui, Yuanchun Huang, Jiacai Yang, William Ba-Thein

**Affiliations:** 1 Shantou-Oxford Clinical Research Unit, Shantou University Medical College, 22 Xinling Road, Jinping, Shantou, Guangdong China; 2 Pediatric Department, First Affiliated Hospital of Shantou University Medical College, Shantou, Guangdong China; 3 Department of Clinical Microbiology, First Affiliated Hospital of Shantou University Medical College, Shantou, Guangdong China; 4 Department of Urology, Second Affiliated Hospital, Third Military Medical University, Chongqing, China; 5 Department of Microbiology and Immunology, Shantou University Medical College, Shantou, Guangdong 515041 China

**Keywords:** Nasal carriage, Bacterial pathogen, Risk factor, Healthy children, China

## Abstract

**Background:**

Nasal colonization with bacterial pathogens is associated with risk of invasive respiratory tract infections, but the related information for Chinese healthy children is scarce.

**Methods:**

This cross-sectional study was conducted with healthy children from 6 kindergartens in the Chaoshan region, southern China during 2011–2012. Nasal swabs were examined for five common bacterial pathogens: *Streptococcus pneumoniae*, *Haemophilus influenzae*, *Haemophilus parainfluenzae*, *Moraxella catarrhalis*, and *Staphylococcus aureus*.

**Results:**

Among 1,088 children enrolled, 79.6 % (866) were target-bacterial carriers, of which 34.4 % (298/866) were positive for ≥2 bacteria species. The most common pathogen in the bacterial carriers was *M. catarrhalis* (76.6 %), followed by *S. pneumoniae* (26.6 %), *S. aureus* (21.8 %), *H. parainfluenzae* (12.7 %), and *H. influenzae* (2.3 %). Multiple logistic regression analyses showed negative associations between age and the overall or multiple bacterial carriage, and between the father’s education level and multiple bacterial carriage (all *p* < 0.05). Age was negatively associated with the carriage of *M. catarrhalis* and *S. pneumoniae*, and positively associated with the *S. aureus* carriage (all *p* < 0.0001).

**Conclusions:**

This study shows high nasal carriage of common pathogenic bacteria and coexistence of multiple pathogens in healthy Chaoshan kindergarten children, with *M. catarrhalis* as the commonest colonizer. Increasing age of children and higher paternal education are associated with lower risk of bacterial carriage. Longitudinal follow-up studies would be helpful for better understanding the infection risk in bacterial pathogen carriers*.*

**Electronic supplementary material:**

The online version of this article (doi:10.1186/s12887-016-0703-x) contains supplementary material, which is available to authorized users.

## Background

Respiratory tract infections (RTIs) are a major cause of death in children worldwide. Although viruses are main RTI pathogens, bacteria are responsible for some localized RTIs such as sinusitis or pneumonia. Globally, *Streptococcus pneumoniae* and *Haemophilus influenzae* are the leading pathogens of RTIs, *S. pneumoniae* caused 0.8 million deaths in children younger than 5 years in 2000 [1], and *H. influenzae* accounted for at least 0.4 million deaths in 2006 [2]. *Moraxella catarrhalis,* along with *S. pneumoniae* and *H. influenzae*, constitutes top three bacterial pathogens causing community-acquired RTIs [3, 4]. *Staphylococcus aureus* is an important cause of infections both in the community and hospital, including pneumonia and neonatal sepsis [5, 6].

Nasopharyngeal colonization with respiratory pathogens is a precursor to the onset of RTI [7]. Most colonization remains asymptomatic, but it can become invasive in susceptible hosts [7]. Bacterial colonization can progress into primary infection or secondary superinfection after viral infection in its host [8]. Besides, asymptomatic carriers are a recognized source of community-acquired RTIs [6, 7, 9].

The carriage of bacterial pathogens is higher in children than in adults [7, 9]. Previous studies have shown that *S. pneumoniae*, *H. influenzae,* and/or *M. catarrhalis* can colonize in children between 1 and 30 months [7], or even as early as 8–10 days after birth [10]. Up to one hundred per cent of infants aged 1 year were reported to carry at least one respiratory pathogens [7]. Multiple carriage of respiratory bacterial pathogens is common in children, particularly in kindergartens [7, 9] because of easier transmission through close contact.

Many studies have reported nasal carriage of bacterial pathogens in children, but most of them focused on one or two pathogens [9]. Little is known about bacterial pathogens in Chinese healthy children. Thus, we have investigated the nasal carriage of five common bacterial pathogens (*S. pneumoniae, H. influenzae*, *H. parainfluenzae*, *M. catarrhalis,* and *S. aureus*) in healthy kindergarten children in China.

## Methods

### Study site and population

In this cross-sectional study, 6 urban/rural kindergartens in the Chaoshan region (Shantou, Jieyang, and Chaozhou cities), Guangdong, southern China were selected through convenient sampling during October 2011 and January 2012. This study period was selected to avoid summer, winter, or national holidays and conflicts with kindergartens’ schedules. The Chaoshan region is in the subtropical zone, with a population of 14.6 million, approximately 2,100 kindergartens and 0.4 million kindergarten children in 2011 [11–13].

Eligible participants were screened 1 week prior to the sample collection by sending a pretested questionnaire (Additional file 1) to the parents/guardians of the kindergarten children to collect the demographic and medical information, including a history of vaccination, respiratory symptoms in the past 6 months, antibiotic consumption within previous 3 months, and immunodeficient conditions. Further, a phone call with parents/guardians was made to reassure the eligibility in the morning of sample collection days.

Included in the study were all children aged 2–6 years old, except those with acute respiratory symptoms (<72 h of the onset), antibiotic consumption within 7 days of enrolment, immunodeficient conditions, and any unfit physical condition for swab collection, or those without consent.

### Sample collection

Our trained staff took a nasal swab from one nostril of each participant as per WHO guidelines [14]. The swab was inoculated immediately onto blood agar and chocolate agar plates on the study site, transported in anaerobic jars (AnaeroPack Rectangular Jar and AnaeroPack-MicroAero, Mitsubishi Gas Chemical Company, Japan) to our laboratory within 1–3 h, and incubated in 5 % CO_2_ at 35 °C for 24–48 h for isolation and identification.

### Microbial isolation and identification

The specimens were tested for the presence of *S. pneumoniae*, *H. influenzae, H. parainfluenzae*, *M. catarrhalis*, and *S. aureus* according to the standard laboratory procedures. *S. pneumoniae* was identified by colony morphology, Gram staining, catalase and α-haemolysis, and optochin susceptibility. *H. influenzae* and *H. parainfluenzae* were identified by growth on chocolate agar with bacitracin, colony morphology, Gram staining, catalase reaction, and requirement of X (hemin), V (nicotinamide adenine dinucleotide), and X + V factors (Oxoid, Basingstoke, the United Kingdom). *M. catarrhalis* was identified by colony morphology, Gram staining, oxidase and DNase tests, and growth on chocolate agar with vancomycin, trimethoprim, amphotericin B and acetazolamide. *S. aureus* was identified by colony morphology, Gram staining, β-haemolysis, and catalase and coagulase tests.

### Statistical analysis

We used Chi-square test to compare differences in the proportions of categorical variables, and multiple logistic regression models to test associations between demographic characteristics and bacterial carriage or to test bacterial coexistence. All the analyses were conducted in SPSS version 17.0, with a two-tailed *p* < 0.05 as significant difference. Missing data were not included in the analyses. Statistical estimates were reported after adjusting for confounders.

## Results

The study participants represented 32.5 % (1,088/3,348) of all children attending 6 kindergartens in 3 cities (6 districts): Shantou (Jinping [42.7 %, 167/391], Chenghai [33.3 %, 202/607], Chaoyang [32.7 %, 180/551], and Longhu [24.6 %, 150/610]), Jieyang (Rongcheng [35.1 %, 196/558]), and Chaozhou (Xiangqiao [30.6 %, 193/631]), or 0.8 % (1,088/0.14 million) and 0.3 % (1,088/0.4 million) of all kindergarten children in the 6 districts and in Chaoshan region, respectively.

Among the children, 79.6 % (866/1,088) were target-bacterial carriers, of which 34.4 % (298/866) were positive for ≥2 bacterial spp. (Table 1). The overall carriage of target bacteria was significantly lower in the oldest age group (5 ~ 6 years) compared to that in the youngest age group (2 ~ <3 years) (OR: 0.3, 95 % CI: 0.2–0.5, *p* < 0.05), and higher in the children from Chaozhou than in those from Shantou (OR: 2.5, 95 % CI: 1.5–4.0, *p* < 0.05, Table 1). The children who were older or who had parents with higher education were less likely to carry multiple bacteria (all *p* < 0.05, Table 1).

**Table 1:** Characteristics of healthy kindergarten children (*n* = 1,088) and carriage of pathogenic bacteria

Independent Variable	Total (*n* = 1,088) n (%)^a^	Bacterial carriage n (%)^b^		OR (95 % CI)^c^	Bacteria carriers (*n* = 866) n (%)^b^		OR (95 % CI)^d^
		No (*n* = 222)	Yes (*n* = 866)		Single spp. (*n* = 568)	Multiple spp. (*n* = 298)	
Gender							
Male	593 (54.5)	122 (20.6)	471 (79.4)	Reference	312 (66.2)	159 (33.8)	Reference
Female	495 (45.5)	100 (20.2)	395 (79.8)	1.0 (0.8–1.4)	256 (64.8)	139 (35.2)	1.1 (0.8–1.4)
Age (years)							
2 ~ < 3	218 (20.0)	31 (14.2)	187 (85.8)	Reference	106 (56.7)	81 (43.3)	Reference
3 ~ < 4	352 (32.4)	61 (17.3)	291 (82.7)	0.8 (0.5–1.3)	181 (62.2)	110 (37.8)	0.8 (0.5–1.2)
4 ~ < 5	331 (30.4)	65 (19.6)	266 (80.4)	0.7 (0.4–1.1)	179 (67.3)	87 (32.7)	0.6 (0.4–0.9)*
5 ~ 6	187 (17.2)	65 (34.8)	122 (65.2)	0.3 (0.2–0.5)*	102 (83.6)	20 (16.4)	0.3 (0.1–0.4)*
Episode of respiratory symptoms in the past 6 months							
0	308 (28.3)	59 (19.2)	249 (80.8)	Reference	155 (62.2)	94 (37.8)	Reference
1 ~ 2	679 (62.4)	148 (21.8)	531 (78.2)	0.9 (0.6–1.2)	350 (65.9)	181 (34.1)	0.9 (0.6–1.2)
≥ 3	101 (9.3)	15 (14.9)	86 (85.1)	1.4 (0.7–2.5)	63 (73.3)	23 (26.7)	0.6 (0.4–1.0)
Antibiotic consumption within previous 3 months (n = 875, 213 missing data)							
No	524 (59.9)	101 (19.3)	423 (80.7)	Reference	279 (66.0)	144 (34.0)	Reference
Yes	351 (40.1)	85 (24.2)	266 (75.8)	0.7 (0.5–1.0)	179 (67.3)	87 (32.7)	0.9 (0.7–1.3)
Mother’s education level (n = 1,059, 29 missing data)							
Elementary school	225 (21.2)	45 (20.0)	180 (80.0)	Reference	100 (55.6)	80 (44.4)	Reference
Middle/high school	592 (55.9)	111 (18.8)	481 (81.3)	1.1 (0.7–1.6)	312 (64.9)	169 (35.1)	0.7 (0.5–1.0)*
College/university	242 (22.9)	56 (23.1)	186 (76.9)	0.8 (0.5–1.3)	143 (76.9)	43 (23.1)	0.4 (0.2–0.6)*
Father’s education level (n = 1,063, 25 missing data)							
Elementary school	186 (17.5)	36 (19.4)	150 (80.6)	Reference	83 (55.3)	67 (44.7)	Reference
Middle/high school	584 (54.9)	106 (18.2)	478 (81.8)	1.1 (0.7–1.6)	298 (62.3)	180 (37.7)	0.7 (0.5–1.1)
College/university	293 (27.6)	70 (23.9)	223 (76.1)	0.8 (0.5–1.2)	176 (78.9)	47 (21.1)	0.3 (0.2–0.5)*
Location of kindergarten (city)							
Shantou	699 (64.2)	162 (23.2)	537 (76.8)	Reference	348 (64.8)	189 (35.2)	Reference
Jieyang	196 (18.0)	39 (19.9)	157 (80.1)	1.2 (0.8–1.8)	106 (67.5)	51 (32.5)	0.9 (0.6–1.3)
Chaozhou	193 (17.7)	21 (10.9)	172 (89.1)	2.5 (1.5–4.0)*	114 (66.3)	58 (33.7)	0.9 (0.7–1.3)

^a^% of total

^b^% of each independent variable

^c^Between-group comparison regarding the overall bacterial carriage by Chi-square test

^d^Between-group comparison regarding multiple bacterial carriage by Chi-square test

^*^ *p* < 0.05

**Fig. 1:**
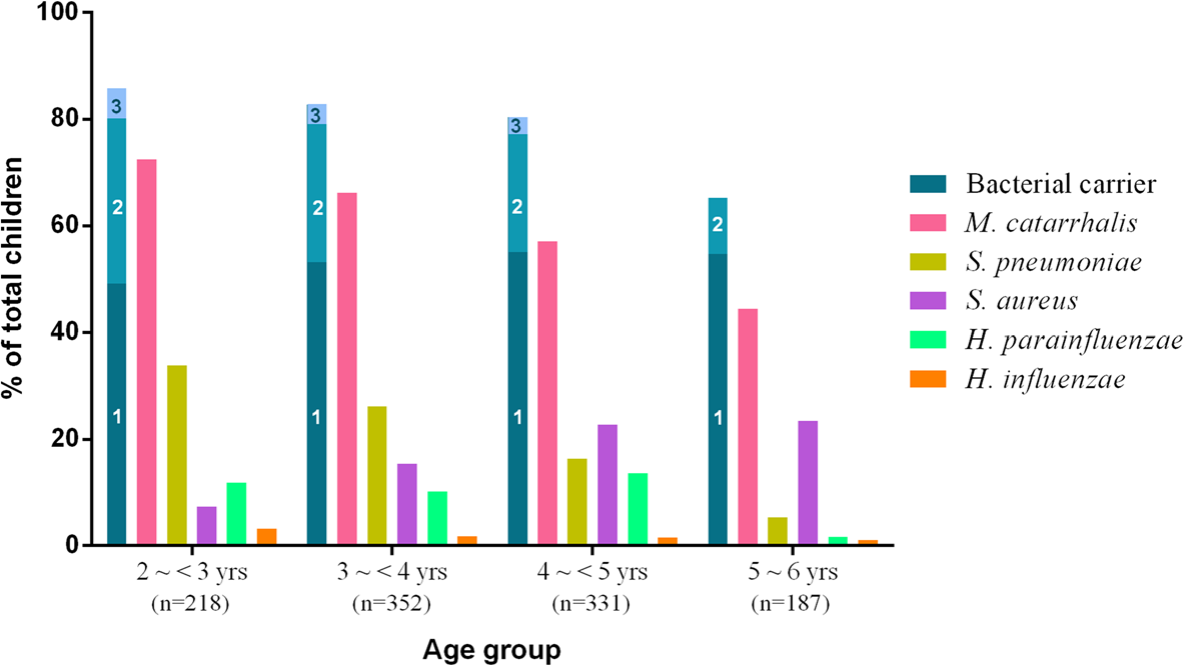
Bacterial carriage in healthy kindergarten children by age group (*n* = 1,088). The cardinal numbers in the first column indicate the no. of bacterial species carried by individual children. Carriage of 4 bacterial species accounted for 0.7 % and 0.8 % of children in the 3 ~ <4 years- and 4 ~ <5 years-age groups, respectively (not shown in figure)

The most common pathogen among the bacterial carriers was *M. catarrhalis* (76.6 %, 663/866), followed by *S. pneumoniae* (26.6 %, 230/866), *S. aureus* (21.8 %, 189/866), *H. parainfluenzae* (12.7 %, 110/866), and *H. influenzae* (2.3 %, 20/866). Single or multiple bacterial carriages decreased with increasing age, except *S. aureus* carriage that increased with age (Figure 1).

Multiple logistic regression analyses (Table 2) showed negative associations between age and the overall carriage (β: −0.3, aOR: 0.8, 95 % CI: 0.7–0.9, *p* < 0.001) or multiple carriage (β: −0.2, aOR: 0.8, 95 % CI: 0.7–0.9, *p* < 0.01) of bacteria, and between the father’s education level and multiple bacterial carriage (β: −1.1, aOR: 0.3, 95 % CI: 0.2–0.5, *p* < 0.0001). Age was negatively associated with the carriage of *M. catarrhalis* (β: −0.4, aOR: 0.7, 95 % CI: 0.6–0.8, *p* < 0.0001) and *S. pneumoniae* (β: −0.4, aOR: 0.7, 95 % CI: 0.6–0.8, *p* < 0.0001), but positively associated with the *S. aureus* carriage (β: 0.4, aOR: 1.5, 95 % CI: 1.3–1.7, *p* < 0.0001, data not shown). Risk of carrying target bacteria was higher among the children in Chaozhou than in Shantou (β: 1.1, aOR: 2.9, 95 % CI: 1.7–4.8, *p* < 0.0001).

**Table 2:** Multiple logistic regression analyses of bacterial carriage in healthy kindergarten children

Variable	Overall bacterial carriage				Multiple bacterial carriage			
	β	aOR	95 % CI	*p* value	β	aOR	95 % CI	*p* value
Increasing age^a^	−0.3	0.8	(0.7–0.9)	<0.001	−0.2	0.8	(0.7–0.9)	<0.01
Father’s education level								
Elementary school	-	-	-	-	Ref.	-	-	-
Middle/high school	-	-	-	-	−0.3	0.8	(0.5–1.1)	0.18
College/university	-	-	-	-	−1.1	0.3	(0.2–0.5)	<0.0001
Location of kindergarten (city)								
Shantou	Ref.	-	-	-	-	-	-	-
Jieyang	0.3	1.3	(0.9–1.9)	0.21	-	-	-	-
Chaozhou	1.1	2.9	(1.7–4.8)	<0.0001	-	-	-	-

*Ref* reference

^a^Continuous variable

Multiple logistic regression analyses (Table 3) showed that *M. catarrhalis* was positively associated with *S. pneumoniae* but negatively with *S. aureus*; *S. pneumoniae* was positively associated with *M. catarrhalis* and *H. parainfluenzae* but negatively with *S. aureus*; *S. aureus* was negatively associated with *M. catarrhalis*, *S. pneumoniae*, and *H. parainfluenzae*; *H. parainfluenzae* was positively associated with *S. pneumoniae* but negatively with *S. aureus* (all *p* < 0.05).

**Table 3:** Multiple logistic regression analyses of bacterial coexistence

Covariate		*M. catarrhalis*	*S. pneumoniae*	*S. aureus*	*H. parainfluenzae*	*H. influenzae*
*M. catarrhalis* (n = 663)	n (%)	-	168 (73.0)	81 (42.9)	66 (60.0)	10 (50.0)
	β		0.6	−1.0	−0.1	−1.0
	aOR (95 % CI)		1.9 (1.3–2.8)*	0.4 (0.3–0.6)*	1.0 (0.6–1.7)	0.4 (0.1–1.2)
*S. pneumoniae* (n = 230)	n (%)	168 (25.3)	-	18 (9.5)	42 (38.2)	3 (15.0)
	β	0.7		−1.2	0.6	−1.2
	aOR (95 % CI)	2.0 (1.3–3.0)*		0.3 (0.2–0.6)*	1.8 (1.0–3.2)*	0.3 (0.1–1.7)
*S. aureus* (n = 189)	n (%)	81 (12.2)	18 (7.8)	-	8 (7.3)	2 (10.0)
	β	−0.9	−1.2		−1.4	−0.2
	aOR (95 % CI)	0.4 (0.3–0.6)*	0.3 (0.2–0.6)*		0.2 (0.1–0.6)*	0.8 (0.1–4.3)
*H. parainfluenzae* (n = 110)	n (%)	66 (10.0)	42 (18.3)	8 (4.2)	-	0 (0)
	β	0.1	0.6	−1.3		−17.8
	aOR (95 % CI)	1.1 (0.6–1.8)	1.9 (1.1–3.3)*	0.3 (0.1–0.7)*		0 (0-NaN)
*H. influenzae* (n = 20)	n (%)	10 (1.5)	3 (1.3)	2 (1.1)	0 (0)	-
	β	−0.9	−0.6	0.1	−19.1	
	aOR (95 % CI)	0.4 (0.1–1.3)	0.6 (0.1–2.8)	1.1 (0.2–5.4)	0 (0-NaN)	

*aOR* adjusted for age, gender, history of RTI symptoms and antibiotic consumption, education levels of parents, and location of kindergartens

^*^ *p* < 0.05

## Discussion

This is the first study demonstrating a high frequency of carriage (79.6 %) of common pathogenic bacteria among healthy kindergarten children in Chaoshan region, southern China.

Nasal carriage rates of bacterial pathogens in healthy preschool or kindergarten children vary widely with studies and geographies. The global carriage rates of *M. catarrhalis* (16–67 %) [15–19], *S. pneumoniae* (10–69 %) [9, 15, 17, 19, 20], and *S. aureus* (10–50 %) [15, 17, 19, 21] are similar to the corresponding rates as 60.9 % (663/1,088), 21.1 % (230/1,088), and 17.3 % (189/1,088) in this study. However, the carriage rates of *H. parainfluenzae* (10.1 %, 110/1,088) and *H. influenzae* (1.8 %, 20/1,088) in this study are respectively much lower than 50.5–86.7 % in other studies from China [15, 21] and 10–83 % globally [15–17, 19]. Previous consumption of antibiotics or Hib vaccination could be the reasons for such low carriage although we could not verify this from incomplete information returned by the parents/guardians.

Predominant bacterial pathogens colonizing in healthy preschool children also differ geographically, with the most prevalent pathogen being *M. catarrhalis* in the present study (60.9 %) and Korea (35 %) [20], *S. pneumoniae* in the Czech Republic (38.1 %) [17], *H. parainfluenzae* in southern China (50.5 %) [21] and southwest China (86.7 %) [15], and *H. influenzae* in Belgium (83 %) [19]. The carriage of *M. catarrhalis, S. pneumoniae*, or *H. influenzae* is generally higher in the low income countries than that in the low-middle income countries [9], whereas the carriage of *S. aureus* is higher in the developed countries than in the developing countries [22].

Similar to our finding (34.4 %), multiple bacterial carriage, which can potentially lead to severe, life-threatening diseases [7, 9], is also common in healthy preschool/kindergarten children globally, for example, 19.8 % in the Czech Republic [17], 31.5 % in Korea [20], and 60.7 % in Belgium [19].

The carriage of these bacteria appears to fluctuate in a dynamic process throughout the host’s lifetime [6, 7, 20], with preschool period considered susceptible to infections due to insufficient innate immunity and close peer contact. Colonization and transmission of bacterial pathogens can be influenced by complex interplay of various factors, such as age, prior respiratory infection, antimicrobial use, and hygiene [7, 9].

In general, the carriage of bacterial pathogens decreases with age [6, 7, 9], with *M. catarrhalis* and *S. pneumoniae* in this study as an example. The colonization of *M. catarrhalis*, *S. pneumoniae*, and *H. influenzae* is higher in preschool children than in school children [20], or in children than in adults [9, 23]. In contrast, the carriage of *S. aureus* increases with age, as seen in this study and in Korea [20].

Although respiratory viral infections and/or antimicrobial consumption can precede bacterial colonization [7, 9, 19], no significant association was observed in this study and other studies as well [24–26].

The education levels of fathers and mothers were strongly correlated in this study (*r* = 0.7, *p* < 0.01 by Spearman’s correlation coefficient, a non-adjusted bivariate correlational analysis, data not shown); in multiple logistic regression models, the education level of fathers (not the mothers) had a positive relationship with multiple bacterial carriage in their children (Table 2), suggestive of better hygiene knowledge from higher education in those families. Poor hygienic conditions could also explain higher risk of bacterial carriage in the Chaozhou kindergarten in this study.

As observed in this study, coexistence or lack of coexistence of certain bacterial pathogens in nasal cavity have been reported in different countries [19, 20, 27, 28]. Particular attention should be paid to the coexistence because of its potential risk of infections, as exemplified by the reports that there are strong relationships between the colonization of *M. catarrhalis, S. pneumoniae*, and/or nontypable *H. influenzae* and otitis media [29, 30]. Screening pathogens in nasal microbiota and observing unwanted outcomes longitudinally may therefore be important in understanding the etiologic role of colonized pathogens.

Kindergartens, a place with risk of infection transmission, are highly regulated in many countries. Chinese kindergartens, including those in this study, are not satisfactory as to the infection control standards, such as lack of physical examination, crowded conditions, limited hygiene knowledge, and improper hygiene practices of staff and children, which are reflected by high isolation rates of bacterial pathogens from the hands of staff and children, inanimate surfaces, and toys [31–33]. The children carrying pathogenic bacteria are a potential source of drug-resistant pathogens, especially in face of prevailing antimicrobial overuse or misuse in Chinese pediatric population [34, 35]. Given carrying bacterial pathogens as a prelude of RTIs and potential of microbial transmission under poor hygienic conditions in crowded kindergartens, the study children are at high risk of RTIs.

There were limitations in this study. Health status of the participants was based on their past medical history provided in the questionnaire. Children and kindergartens in this study represented a relatively small proportion of those in the Chaoshan region. Therefore, our results should be interpreted cautiously. Intrinsic to cross-sectional study design, true cause and effect relationships between bacterial carriage and the factors investigated could not be established. Further studies are needed to understand the influence of bacterial carriages and disease occurrence.

## Conclusions

This study demonstrates high nasal carriage of common pathogenic bacteria and coexistence of multiple pathogens in healthy kindergarten children in the Chaoshan region of southern China, with *M. catarrhalis* as the commonest colonizer. Increasing age of children and higher paternal education are associated with lower risk of bacterial carriage. Longitudinal follow-up studies would be required for better understanding the infection risk in bacterial pathogen carriers.

## Additional file

Additional file 1: Questionnaire. A questionnaire used for demographic/medical data collection from the kindergarten children and their parents/guardians. (DOCX 20 kb)
