# Supplementary material for: Nasal carriage of common bacterial pathogens among healthy kindergarten children in Chaoshan region, southern China: a cross-sectional study
Source: BMC Pediatr. 2016 Sep 30;16:161. doi: 10.1186/s12887-016-0703-x (PMC5064895; doi:10.1186/s12887-016-0703-x)
Supplement: Additional file 1: — Questionnaire. A questionnaire used for demographic/medical data collection from the kindergarten children and their parents/guardians. (DOCX 20 kb) [file 12887_2016_703_MOESM1_ESM.docx]

**Carriage of respiratory pathogens in healthy kindergarten children**

Serial # : **__________________**

**GENERAL INFORMATION**

1. **Location: Shantou___ Chaozhou ___ Jieyang ___**
2. **Name of kindergarten____________**
3. **Size of the class: （please fill in with the number of students in your child’s class）**
4. **Sex: Male ____ Female____**
5. **DOB (dd/mm/yyyy): ______/_______/_________**

**OTHER RELEVANT INFO.**

1. **Vaccination: please check your child’s vaccination record and tick in the following box**

| **Planned vaccination** | **Additional vaccination** |
| --- | --- |
| **Hepatitis B Vaccine ( )** | **Influenza Vaccine ( )** |
| **Bacillus Calmette-Guerin ( )** | **Pneumococcal vaccine ( )** |
| **Poliomyelitis ( )** | **Hib vaccine ( )** |
| **Measles vaccine ( )** | **Rotavirus vaccine ( )** |
| **A/C meningococcal vaccine ( )** | **Rabies vaccine ( )** |
| **Japanese B encephalitis vaccine ( )** | **Chickenpox Vaccine ( )** |
| **Hepatitis A vaccines ( )** | **Unknown ( )** |

1. **History of respiratory infections within the preceding 6 months?**

| **time** | **Number of episodes of respiratory infections** |
| --- | --- |
| **One month ago** |  |
| **Two months ago** |  |
| **Three months ago** |  |
| **Four months ago** |  |
| **Five months ago** |  |
| **Six months ago** |  |

1. **Any history of hospitalization? Yes___ No___ Unknown___**

**If “Yes”, please state in the follow table**

| **Date(Month/Year)** | **duration** | **Diagnosis** |
| --- | --- | --- |
|  |  |  |
|  |  |  |
|  |  |  |

1. **Recent antibiotic consumption? Yes___ No___ Unknown___**

**If “YES”, what is it/are they? ___**

| **Date(Month/Year)** | **Antibiotics** | **Reasons** | **How long (in days)** |
| --- | --- | --- | --- |
|  |  |  |  |
|  |  |  |  |
|  |  |  |  |

1. **Are there any family member working as healthcare personnel?**

**Yes___ No___ Unknown___ If “Yes”, please state in the following box**

| **Relationship to the child** | **Occupation** |
| --- | --- |
|  |  |
|  |  |
|  |  |

1. **Are there any family member being hospitalized in the past month?**

**Yes___ No___ Unknown___ If “Yes”, please state in the following box**

| **Relationship to the child** | **Date of admission(Month/Year)** | **diagnosis** |
| --- | --- | --- |
|  |  |  |
|  |  |  |
|  |  |  |

1. **Exposure to in-house passive smoking: Yes___ No___ Unknown___**
2. **Current living space:**

**＜60M^2^ ___ 61-90M^2^ ___ 91-120 M^2^ ___ 121-150 M^2^ ___ ＞150 M^2^___**

1. **Number of people living in the household: _______ persons**
2. **Having older siblings: Yes___ No___ if “YES”, please list their age in the following box (one child per box, state age in years)**

|  |  |  |  |  |  |
| --- | --- | --- | --- | --- | --- |

1. **Mother’s education: Post-graduate ___ Graduate ___High school___ Primary school___**
2. **Father’s education: Post-graduate ___ Graduate ___High school___ Primary school___**
3. **Family income per month (in RMB):**

**＞10000___ 6000-10000___3000-6000___1000-3000___＜1000___**

**---------------------------------------------------------------------------------------------------------------------------**

**RESEARCH STAFF USE ONLY**

**SPECIMEN COLLECTION**

1. **Does the child meet the inclusion/exclusion criteria? Yes___ No___**

**If no (do not take swab), state reason: _______________________________________**

1. **Any condition preventing the collection of swab (e.g., nasal polyps): Yes___ No___ Unknown___**

**If YES, what is it?_________________________________**

1. **Specimen collected: Nasal swab (left) ___ (right) ___**

**Name of the staff obtaining the nasal swab: ______________________**

**Name of the staff inoculate the plates: ______________________**

**Name of the staff taking this record: ______________________**

**Date: ______/_______/_________**
